# Enhanced Fermentation of Pu-Erh Tea with *Aspergillus niger*: Quality and Microbial Community Analysis

**DOI:** 10.3390/molecules29235647

**Published:** 2024-11-28

**Authors:** Jingchuan Zheng, Lijun Yu, Muhammad Aaqil, Qiaomei Wang, Wenshu Peng, Li Zhuang, Wanying Gong, Tingting Zheng, Miaomiao Zhao, Chao Wang, Xingjiao Jiang, Liang Yan, Ruijuan Yang

**Affiliations:** 1 College of Food Science and Technology, Yunnan Agricultural University, Kunming 650201, China; 18788190140@163.com (J.Z.); yulijun0519@163.com (L.Y.); aaqilkhan26@gmail.com (M.A.); gongwanying1206@163.com (W.G.); yunnantt@126.com (T.Z.); 13981685983@163.com (C.W.); 18206706425@163.com (X.J.); 2 College of Longrun Pu-erh Tea, Yunnan Agricultural University, Kunming 650201, China; wqm19850127@163.com; 3 College of Tea (Pu’er), West Yunnan University of Applied Sciences, Pu’er 665000, China; xiaomi740@163.com (W.P.); 15891966450@163.com (M.Z.); 4 Pu’er Institute of Pu-erh Tea, Pu’er 665000, China; 5 College of Tea and Coffee, Pu’er University, Pu’er 665000, China; zhuangli@peu.edu.cn

**Keywords:** *Aspergillus niger*, enhanced fermentation, Pu-erh tea, microorganism, food quality

## Abstract

Post-fermented Pu-erh tea (PFPT) is a microbial fermented tea characterized by unique sensory attributes and multiple health benefits. *Aspergillus niger* is the dominant fungus involved in the fermentation process and plays a significant role in imparting the distinct characteristics of PFPT. To investigate the role of *Aspergillus niger* in the fermentation of Pu-erh tea, this study inoculated unsterilized sun-dried green tea with *Aspergillus niger* isolated from Pu-erh tea to enhance the fermentation process. Metabolites and microbial communities in sun-dried green tea (CK), fortified fermented tea (TF), and naturally fermented tea (NF) were analyzed using non-targeted metabolomics, 16S rDNA, and internal transcribed spacer sequencing. Non-targeted metabolomics revealed that *Aspergillus niger* significantly altered the metabolite profile of the tea samples, identifying a total of 200 different metabolites, with 95 showing significant increases and 105 significant decreases, predominantly enriched in metabolic pathways associated with amino acid biosynthesis and degradation. High-throughput sequencing revealed that although the relative abundance of the fungal community remained largely unchanged, the inoculation of *Aspergillus niger* significantly increased the abundance of *Bacillales* and *Pseudomonas* within the bacterial community, thereby influencing the dynamic balance of the microbial ecosystem. Collectively, the inoculation of *Aspergillus niger* altered the composition of the microbial community and metabolic activities, resulting in changes to the content of amino acid-dominated metabolites, thereby enhancing the flavor profile and overall quality of Pu-erh tea. These findings provide important insights for optimizing the production processes of Pu-erh tea and the application of microorganisms in other fermented foods.

## 1. Introduction

Pu-erh tea is made from large-leaf tea [(*Camellia sinensis*.) var. *assamica* (Masters) Kitmaura] as raw material [1], and the sun-dried green tea is processed by post-fermentation, which has a strong soup color, mellow flavor, and obvious characteristics of aging aroma [2]. Pu-erh ripe tea has a unique flavor and good health functions because of its unique post-fermentation process. Post-fermentation is the key to Pu-erh ripe tea processing, and it is the core step in the formation of its quality characteristics and health functions [1]. The post-fermentation process mainly relies on microorganisms in the environment [3,4], and microorganisms that are suitable for growing and reproducing in the environment can produce a variety of enzymes, which contribute to the formation of the unique qualities of Pu-erh tea [5]. As far as the current actual situation is concerned, the traditional Pu-erh tea production is in an open environment, which often suffers from the problems of complex and uncontrollable microbial sources [6], which leads to unstable product quality, and therefore there is an urgent need to improve the traditional Pu-erh tea fermentation process.

Enhanced fermentation is the access of one or more exogenous microorganisms to unsterilized raw materials for fermentation, with a view to improving the quality of fermented products [7]. For example, the enhanced fermentation of Sichuan pickled vegetable by lactic acid bacteria and yeast can significantly increase the total acid and total ester content thus improving the flavor of the product [8]. The enhanced fermentation of soy sauce by the inoculation of yeast can increase the number of flavor substances and improve the organoleptic quality [9]. At present, it has been found that the inoculation of bacteria-enhanced fermentation can shorten the processing time of Pu-erh tea, reduce the cost, and improve the sensory quality of tea [10]. Inoculation with *Bacillus licheniformis L*1 fermented Pu-erh tea can significantly increase the quality scores of theaflavin, theobromine, and soluble sugars in the tea broth [11]; inoculation with *Branched Ploughmill A*1 fermented Pu-erh tea increased the scores of sweetness and heaviness in tea broth, and improved the sensory quality [12]. Current research indicates that the dynamic changes in microbial communities during Pu-erh tea fermentation are closely associated with the transformation of its chemical components. These changes not only shape the sensory characteristics of Pu-erh tea but also play a significant role in the generation of its functional compounds. However, most studies on Pu-erh tea fermentation focus primarily on describing microbial diversity, with limited exploration into the regulatory mechanisms of microbial communities and their impact on metabolites.

*Aspergillus niger* is a common mold that is widely distributed in nature and is especially common in grain, plant products, and soil. As an important fermentation industrial strain, it has vigorous growth, a short fermentation cycle, no toxin production, is one of the FDA certified safe strains (GRAS), and is also an important enzyme production strain. *Aspergillus niger* can produce amylase, cellulase, pectinase, glucose oxidase, citric acid, gluconic acid, and gallic acid as well as other pleasant flavor substances during the fermentation process. In traditional fermentation, *Aspergillus niger* is a high yielding strain for the industrial production of cellulase, xylanase, protease, etc. In fermented foods, *Aspergillus niger* plays an active role in the production of tea, cheese, tofu, and soy sauce. The authors of [13] found that *Aspergillus niger* fermentation can improve the functional properties of corn dietary fiber, and that the addition of *Aspergillus niger* during fermentation of kelp soya sauce resulted in a significant increase in its amino acid content and improved the flavor of the product [14]. Studies have shown that *Aspergillus* species are the most common fungi during the post-fermentation process of Pu-erh tea and in its commercial products [5]. *Aspergillus* isolated from Pu-erh tea exhibits significant fermentation functions. Studies have demonstrated that *Aspergillus* can convert tea polyphenols into bioactive theabrownins [15] and degrade caffeine [16] during fermentation. Moreover, *Aspergillus* is considered a key aroma-producing microorganism in the early stages of fermentation [17], playing a crucial role in the formation of Pu-erh tea’s flavor profile. These findings suggest that *Aspergillus niger* not only facilitates the decomposition of polyphenols and cellulose by secreting various enzymes (e.g., cellulase, polyphenol oxidase) but also plays a pivotal role in the synthesis of aromatic compounds. However, it remains unclear how *Aspergillus niger* influences tea quality by regulating microbial community structures and metabolic pathways. The specific mechanisms underlying this process require further in-depth investigation. Our previous study [18] found that *Aspergillus niger* is a dominant fungus in the fermentation process of Pu-erh tea, which may play an important role in the formation of Pu-erh tea quality. *Aspergillus niger* demonstrates significant potential for enhancing the fermentation of Pu-erh tea. Based on this, we hypothesize that *Aspergillus niger* improves tea quality by regulating the dynamics and balance of microbial communities while promoting the activity of key metabolic pathways. To validate this hypothesis and uncover the underlying mechanisms, this study aims to further investigate the regulatory effects of *Aspergillus niger* on microbial communities and metabolic networks during the fermentation process of Pu-erh tea.

In this study, *Aspergillus niger* isolated and identified from Pu-erh tea, was inoculated into non-sterilized sun-dried green tea for enhanced fermentation. Using high-throughput sequencing and untargeted metabolomics analysis, the regulatory effects of *Aspergillus niger* inoculation on microbial communities and its influence on the composition of tea metabolites was systematically investigated. The aim is to evaluate the feasibility of using *Aspergillus niger* for enhanced inoculation fermentation of Pu-erh tea and to further explore the relationship between the chemical composition of tea and the structure of microbial communities. This research enriches the theoretical understanding of the interactions between microorganisms and metabolites during Pu-erh tea fermentation, provides scientific insights into the mechanisms underlying tea quality formation, supports the improvement of Pu-erh tea production processes and quality control, and offers valuable references for studies in other fermented food domains.

## 2. Results

### 2.1. Metabolite Distribution Profile

**Figure 1:**
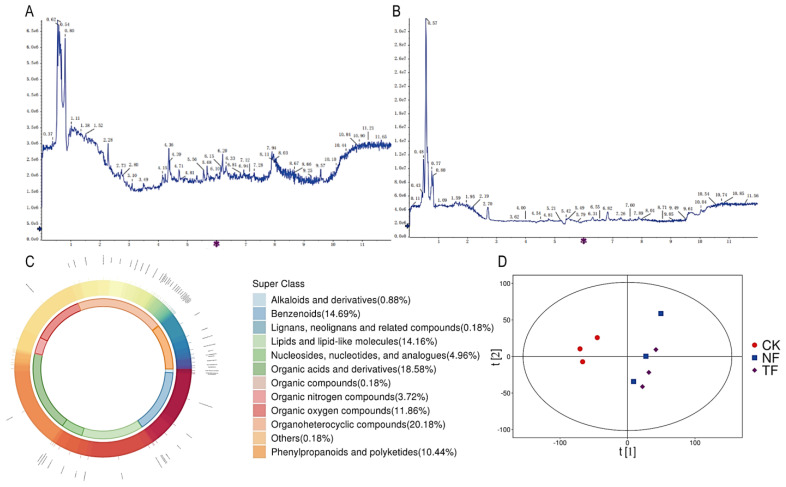
Metabolomics analysis of Pu-erh tea with different fermentation methods. (**A**) Positive ion pattern of total ion flow map of sample. (**B**) Negative ion pattern of total ion flow map of sample. (**C**) Metabolite classification map. (**D**) PCA.

In this study, UPLC-MS/MS metabolomics were used to characterize the overall chemical properties of tea samples before and after fermentation. The total ion current (TIC) overlap plots of mass spectrometry in positive and negative ion modes were obtained by detection (Figure 1A,B), which showed that the TIC curves of the secondary metabolites had high overlap, the retention times and peak intensities were consistent, and the cross contamination between the samples was in the acceptable range, with high stability of the instrument, and high reproducibility and reliability of the data. A total of 6349 compound ions were obtained for subsequent analysis after peak matching and peak area calibration. A total of 565 compounds were identified by comparison with standards, primary mass spectrometry and secondary mass spectrometry analysis (Table S1), including 67 amino acid compounds, 50 fatty acid compounds, 34 saccharides, 26 alkaloids, 25 flavonoids, 24 organic acids and their derivatives, 22 organic heterocyclic compounds, 13 aromatic compounds, and 134 other compounds (Figure 1C).

The raw state of metabolite data can be reflected by using PCA. PCA of the metabolites of Pu-erh tea from different fermentation methods showed (Figure 1D) that the two main components (PC1 and PC2) explained 37.4% and 16.1% of the total variance, respectively, and that CK had a significant tendency to be separated from NF and TF in the principal component scoring plots, while NF and TF were closer but distinguishable in the principal component scoring plots. The results showed that the metabolites of CK were more different from those of NF and TF, while the metabolites of NF and TF were less different. This may be because the dominant fungus in tea fermentation is *Aspergillus niger*, resulting in the effect of *Aspergillus niger* addition on tea fermentation being focused on a specific aspect without causing differential changes in the other substances in the tea.

### 2.2. Screening for Differentially Expressed Metabolites

**Figure 2:**
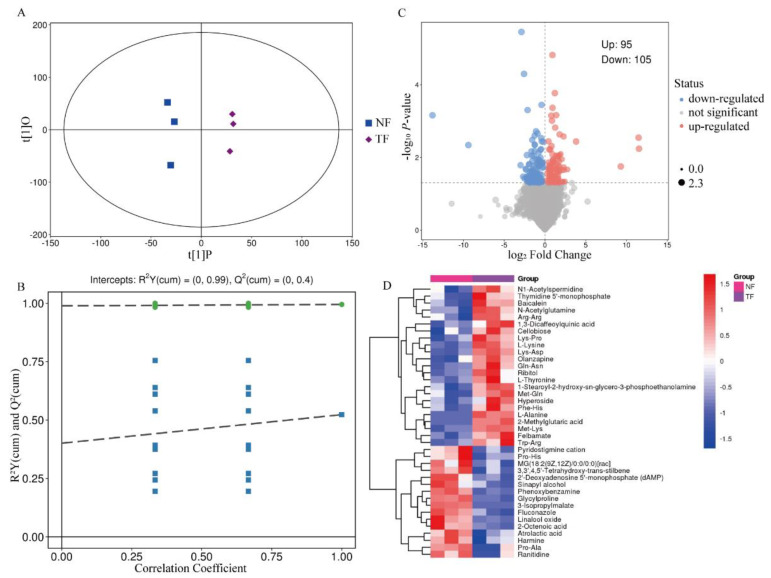
Screening of differential metabolites of Pu-erh tea by different fermentation methods. (**A**) NF vs. TF OPLS-DA score plot. (**B**) NF vs. TF OPLS-DA replacement test plot. (**C**) NF vs. TF differential metabolite volcano plot. (**D**) NF vs. TF differential metabolite matchstick plot (top ten).

Although the principal component analysis method can effectively extract the main information, it is not sensitive to the variables with small correlation. To screen the differently expressed metabolites (DEM) of Pu-erh tea with different fermentation methods, OPLS-DA analysis was performed with all the relevant metabolites as variables. The results showed that both NF and TF were located within the confidence intervals and were clearly differentiated, and the R2Y and Q2 values of the model were high, with a high degree of model explanation, and were stable and reliable (Figure 2A,B), suggesting that there were significant differences between NF and TF metabolites. Screening their DEMs according to VIP > 1.00 and *p* < 0.05 revealed a total of 200 DEMs for NF and TF, 95 metabolites were up-regulated and 105 metabolites were down-regulated (Figure 2C), of which amino acid and fatty acid analogs were more different in the two groups of samples, and amino acid analogs were significantly increased in the samples fermented by *Aspergillus niger* (Table S2) (Figure 2D).

### 2.3. KEGG Metabolic Pathway Enrichment Analysis

**Figure 3:**
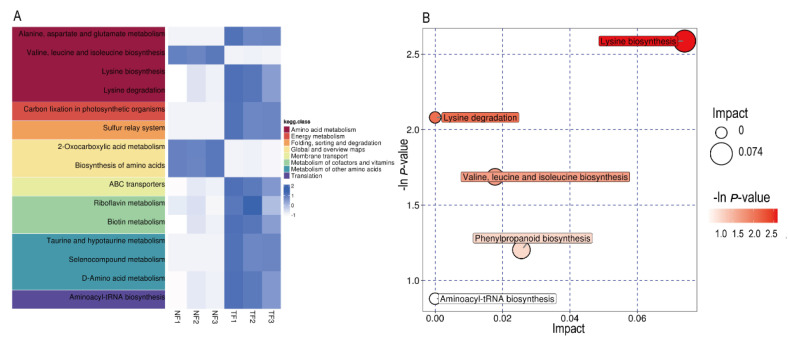
Enrichment analysis of KEGG metabolites in Pu-erh tea with different fermentation methods. (**A**) NF vs. TF KEGG heat map. (**B**) NF vs. TF pathway enrichment bubble map.

All the differential metabolites were matched with the KEGG database to obtain the pathway information of the metabolites, and a total of 24 metabolic pathways were annotated. The heat map analysis of the obtained metabolic pathways revealed that 7 of the top 15 metabolic pathways were directly related to amino acid metabolism (Figure 3A). Enrichment analysis was performed on the annotated metabolites, and the top five metabolic pathways enriched with more differential metabolites were shown in bubble plots (Figure 3B). It was found that the top five KEGG pathways that were the most important in the NF vs. TF group when combined with enrichment and topology analyses were as follows: lysine biosynthesis, lysine degradation, valine, leucine and isoleucine biosynthesis, phenylpropanoid biosynthesis, and aminoacyl-tRNA biosynthesis. The amino acid metabolic pathways were highly enriched by the addition of *Aspergillus niger* on tea fermentation, which is consistent with the results of the differential metabolite analysis within the NF and TF groups.

### 2.4. Microbial Community Analysis

**Figure 4:**
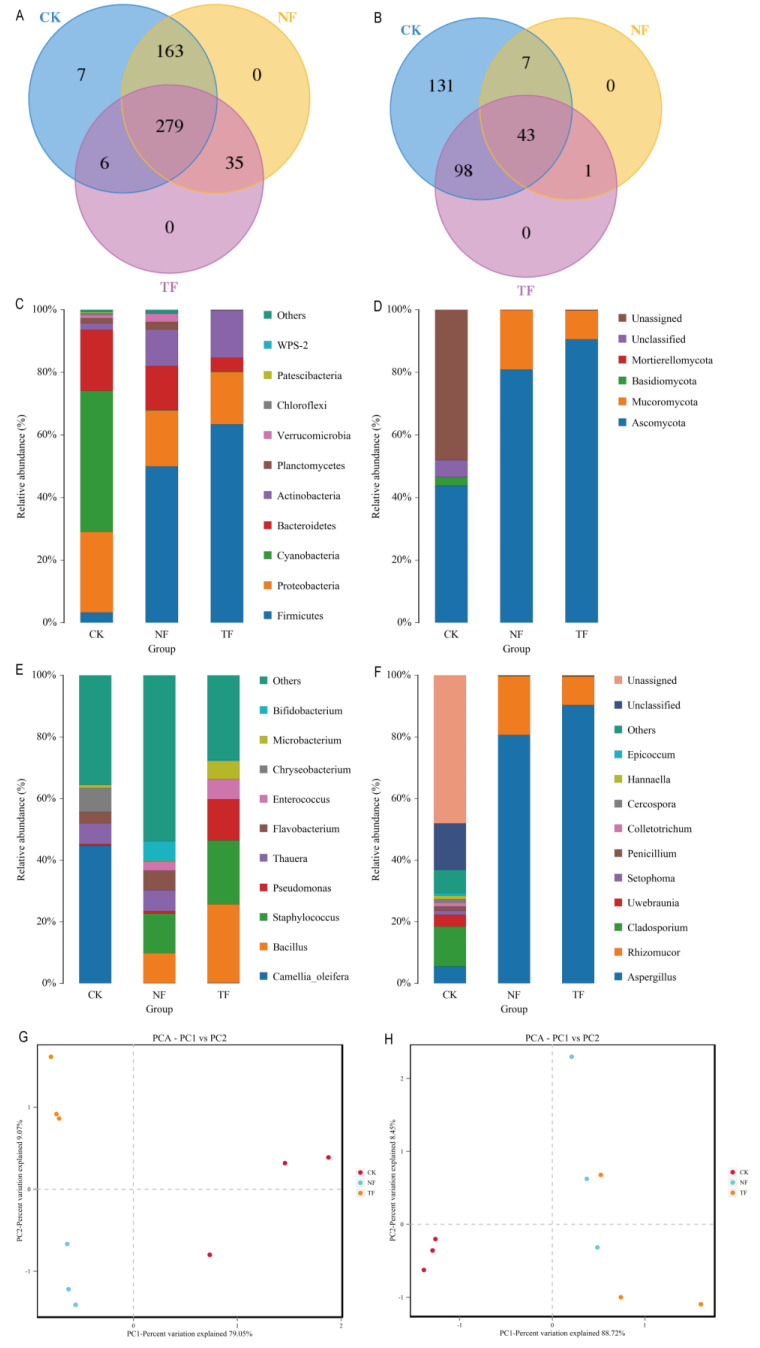
Analysis of microbial composition of samples with different fermentation treatments. (**A**) Bacterial Wayne diagram analysis. (**B**) Fungal Wayne diagram analysis. (**C**) Bacterial community composition at phylum level. (**D**) Fungal community composition at phylum level. (**E**) Bacterial community composition at genus level. (**F**) Fungal community composition at genus level. (**G**) Bacterial community principal component analysis. (**H**) Fungal community principal component analysis.

Considering the fermentation characteristics of Pu-erh tea, the metabolite profiles were closely related to its microbial community. High-throughput sequencing of microorganisms in tea from different fermentation methods was performed based on the V3-V4 region of the bacterial macrogenome 16SrRNA and the ITS1 region of the fungal macrogenome, which, after removing the primers and low-quality ends, combining paired reads, and de-multiplexing, provided at least more than 116,338 bacteria per sample (1,418,304 in total) and 150,275 fungal valid sequences (1,626,751 in total). The sequenced sequences were clustered according to 97% sequence similarity after quality control to obtain 490 bacterial OTUs and 280 fungal OTUs. After counting the number of OTUs among the different samples, it was found that the number of bacterial OTUs increased while the number of fungal OTUs decreased in fermented tea compared with unfermented tea, and the number of TF bacterial OTUs was lower than that of NF, while the number of fungal OTUs was higher than that of NF compared with the different fermentation methods higher than NF (Figure 4A,B).

Microbial annotation results showed that, in terms of bacteria, Cyanobacteria had a high abundance of 45.04% in CK (Figure 4C), but its abundance in NF and TF was as low as less than 1%, and the trend of Proteobacteria and Bacteroidetes was similar to that of Cyanobacteria, with abundance decreasing after tea fermentation. The abundance of Firmicutes was only 3.31% in CK, while the relative abundance of Firmicutes increased to 49.91% and 63.40% in NF and TF, and the trend of Actinobacteria was similar. As for fungi, only four phyla were identified, of which Ascomycota accounted for more than 40% of the sequences of each sample (Figure 4D). In addition, Mucoromycota was mainly present in the fermented tea samples, and the abundance of Mucoromycota in TF was 10% lower compared to that in NF.

At the genus level (Figure 4E), *Camellia_oleifera* was the main dominant genus in CK, followed by *Chryseobacterium*, *Thauera*, *Flavobacterium*, and *Microbacterium*. After fermentation, the relative abundance of *Camellia_oleifera*, the optimal genus in CK, was significantly reduced to 0.18% and 0.19% in NF and TF, whereas the relative abundance of *Bacillus*, *Staphylococcus*, and *Enterococcus* was significantly increased as the dominant genera in NF and TF. It is worth noting that *Thauera*, *Flavobacterium*, and *Bifidobacterium* were also the dominant genera in NF, while *Pseudomonas* and *Microbacterium* were the dominant genera in TF. In terms of fungi, *Cladosporium* was the most abundantly classified and assigned genus in the CK group (Figure 4F). However, after fermentation, *Aspergillus* and *Rhizomucor* were the only dominant genera in NF and TF, the composition of the fungal genera in the post-fermentation samples was more homogeneous, and the relative abundance of *Aspergillus* in TF increased by about 10% compared with that in NF, which indicated that the addition of *Aspergillus niger* changed the composition of fungal genera in the post-fermentation sample.

To further investigate the differences in microbial community structures between different fermentation methods, principal component analysis (PCA) was used for (Figure 4G,H) analysis. Figure 4G and Figure 4H, respectively, illustrate the principal component analysis of bacterial and fungal communities. The PCA models explained 88.12% and 97.17% of the total variance for bacteria and fungi, respectively, indicating good explanatory power. In Figure 4G, the three sample groups are distributed in distinct quadrants with clear separation, suggesting significant differences in bacterial composition between fermentation methods. In contrast, Figure 4H shows no obvious separation between TF and NF, indicating minimal differences in fungal community composition between the two. These results collectively suggest that bacterial community structure is more strongly influenced by the addition of *Aspergillus niger*. The α-diversity analysis showed (Table 1) that there were significant differences in the α-diversity indices between CK, TF, and NF, regardless of fungi or bacteria, and that there were differences in the abundance and diversity of the microbial communities of the three different sample groups. Comparing CK with TF and NF, it was found that the abundance and diversity of both bacteria and fungi were higher in the unfermented samples than in the fermented samples, while comparing TF with NF, it was found that the abundance of bacteria was lower in TF than in NF, while the abundance of fungi was higher than in NF, but the diversity of bacteria and fungi was lower in TF than in the samples from NF.

### 2.5. Differential Analysis of Microbial Communities in Different Fermentation Methods

**Figure 5:**
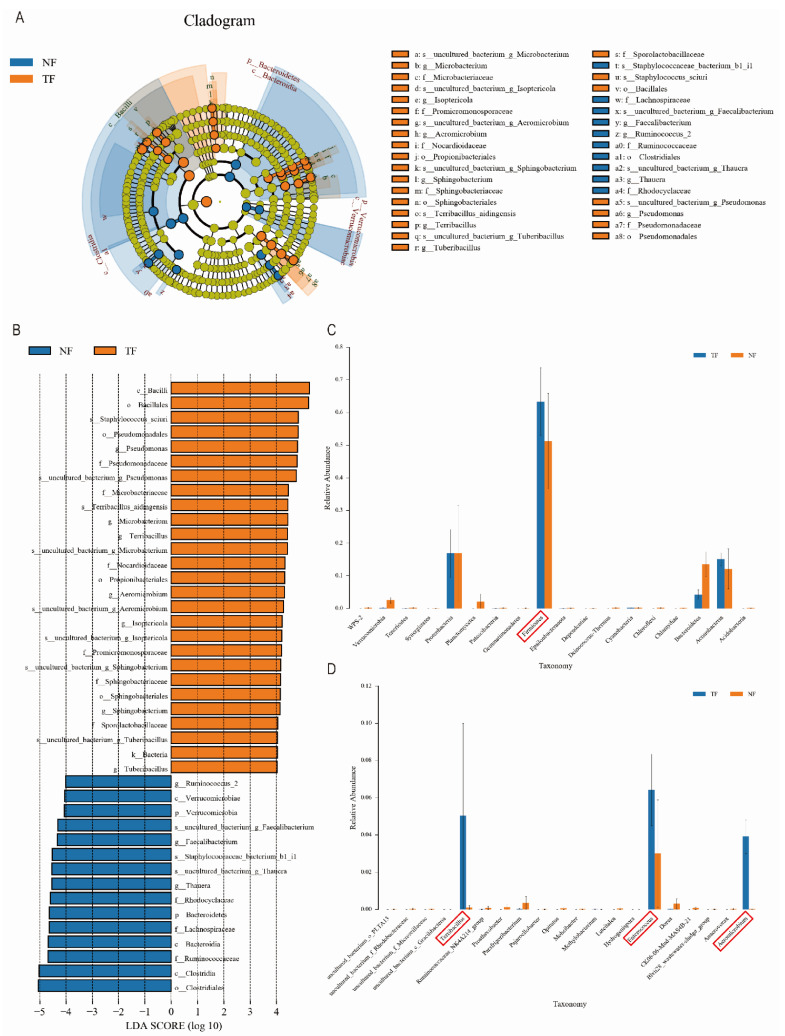
Microbial analysis of differences among groups of different fermentation methods. (**A**) Bacterial LEfSe analysis. (**B**) Bacterial LDA analysis. (**C**) Bacterial phylum level ANOVA analysis. (**D**) Bacterial genus level ANOVA analysis.

Biomarkers in the microbial community were identified by using LEfSe analysis combined with LDA with *p* < 0.05 and LDA > 4 as screening criteria, and the results showed that there was a significant difference in the bacteria in the tea samples after the enhanced fermentation of *Aspergillus niger* (Figure 5A,B), whereas the composition of the fungal community in the TF and the NF, consisting of Ascomycota and Mucoromycota, did not show a significant difference. Bacilli, Staphylococcus-sciuri, Pseudomonadales, and Microbacteriaceae were the most abundant bacterial species in TF, whereas in NF, Clostridia, Ruminococcaceae, Bacteroidia, and Verrucomicrobiae were significantly enriched. Notably, the abundance of Firmicutes significantly increased during fermentation in the TF group (Figure 5C), far surpassing the growth of other microbial communities. Further analysis of the bacterial composition at the genus level revealed that the TF group exhibited high abundances of *Terribacillus*, *Enterococcus*, and *Aeromicrobium* (Figure 5D). Typically, these microbial biomarkers can reflect differences between various fermentation methods and the resulting tea products.

### 2.6. Multivariate Statistical Analysis of Differential Metabolites and Microorganisms

**Figure 6:**
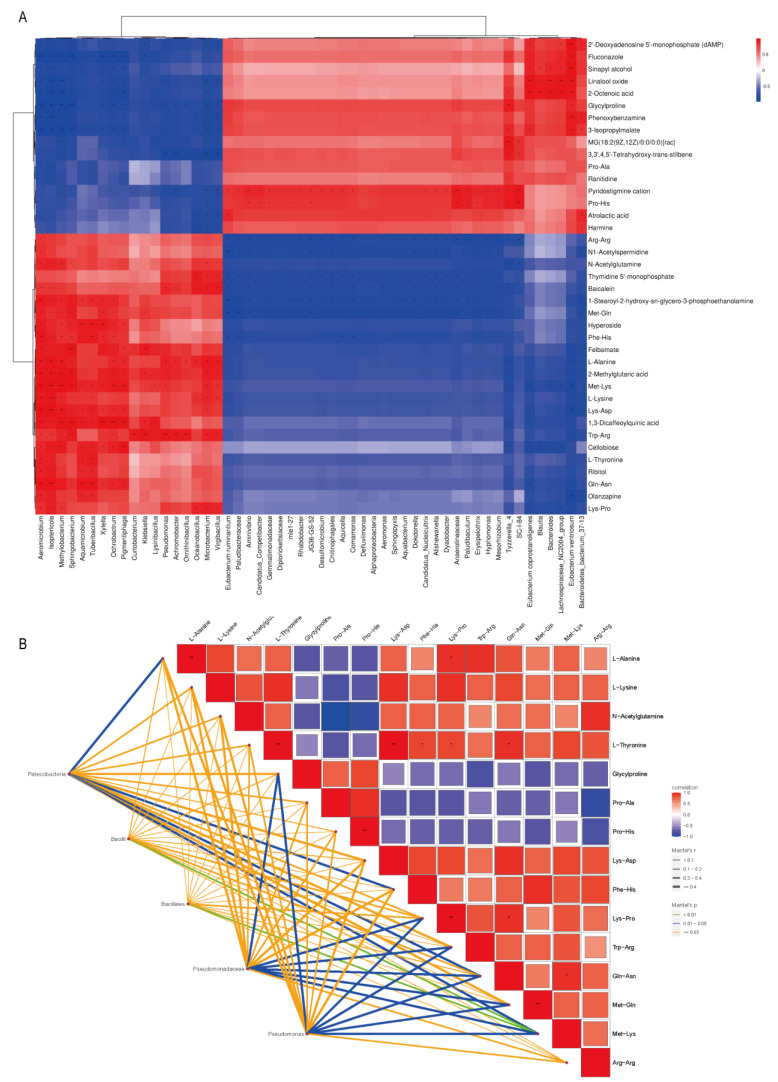
Multivariate statistical analysis of differential metabolites with microorganisms. (**A**) Heat map analysis of differential metabolite association with genus level differential microorganisms based on Euclidean algorithm. (**B**) Heat map analysis of flavor differential metabolite correlation with Mantel test of differential microorganisms at each taxonomic level. * represents *p* < 0.05; ** represents *p* < 0.01; *** represents *p* < 0.001.

Based on the correlation analysis between differential metabolites and differential microorganisms (Figure 6A), a total of 2067 pairs of genus–metabolite relationships were analyzed between differential metabolites and differential microorganisms in *Aspergillus niger* and naturally fermented Pu-erh teas, of which 581 pairs had significant correlation with *p* < 0.05, of which 253 pairs of colony–metabolites were found to be positively significantly correlated with the DEM, and 328 pairs of colony–metabolites were negatively significantly correlated with the DEM. *Aeromicrobium* and *Isoptericola* were both significantly different from 31 kinds of DEM, most of the differential microorganisms showed significant positive correlation with the DEM, and the metabolite Met-Lys was highly different from most of the differential microorganisms in the correlation analysis. Combined with the microbial community difference analysis, the Mantel test correlation heat map analysis of the best combination of content and significance of difference at the level of phylum, class, order, family, and genus with the flavor substances of DEM revealed that (Figure 6B) the flavor substances of *Bacillales* in *Bacilli* and *Pseudomonas* in *Pseudomonadaceae* were significantly correlated with the flavor substances of Pu-erh tea after the enhanced fermentation of *Aspergillus niger*. Specifically, *Bacillales* exhibits an extremely significant correlation with Met-Lys (*p* < 0.01), indicating its key role in protein degradation and dipeptide formation, which directly affects the umami taste and basic flavor of the tea. *Pseudomonas*, on the other hand, is significantly correlated with Glycylproline, Lys-Pro, Trp-Arg, Gln-Asn, Met-Gln, and Met-Lys (*p* < 0.05), highlighting its crucial role in the generation of aromatic compounds thereby enhancing the aroma levels and complexity of the tea. Collectively, this suggests that the metabolic interactions between *Bacillales* and *Pseudomonas* during the *Aspergillus niger* process enhanced the fermentation process jointly to determine the significant changes in the flavor profile of Pu-erh tea.

## 3. Discussion

### 3.1. Enhanced Fermentation by Aspergillus Niger Significantly Alters Microbial Community Structure and Abundance of Bacillales and Pseudomonas in Tea Samples

The interaction between microorganisms and tea quality in post-fermented Pu-erh tea (PEPT) has received increasing attention [19]. Previous studies have shown that the addition of exotic microorganisms not only alters the chemical composition of PEPT but also affects the structure of the microbial community in PEPT [20]. Macrogenomic analyses revealed that the structure of the microbial community in the tea samples and the abundance of some microbial species were altered after the addition of *Aspergillus niger* (Figure 4 and Figure 5). Ref. [11] found that the inoculation of *Penicillium chrysogenum* during the fermentation of Pu-erh tea changed the composition of the fungal community. Ref. [21] found that the addition of *Aspergillus niger* to the pure culture of Pu-erh tea resulted in the production of enzymes that modified the concentration and composition of metabolites in the tea.

In our comprehensive analysis of the microbial communities across the three sample groups, we observed that *Aspergillus niger* occupied a more substantial ecological niche during enhanced fermentation thereby inhibiting bacterial growth and leading to a significant decrease in their abundance. The metabolic byproducts of *Aspergillus niger* supplied essential nutrients for other fungi thereby promoting their synergistic growth and consequently leading to an increase in fungal abundance. Furthermore, microbial abundance and diversity in the *Aspergillus niger*-enhanced fermentation group were significantly diminished, indicating that the emergence of dominant species adversely impacted the richness of other populations and transformed the structure and competitive dynamics within the microbial ecosystem [22]. It has also been found that *Aspergillus niger*-enhanced fermentation resulted in significant alterations to the microbial community, particularly with an increase in the abundance of Firmicutes and Actinobacteria. In contrast, the Cyanobacteria, Proteobacteria, and Bacteroidetes present in pre-fermentation samples were replaced by more resilient or fermentation-adapted species, due to the changes in the microenvironment during the later stages of fermentation. Simultaneously, in terms of fungi, Ascomycota dominated, particularly in the *Aspergillus niger*-enhanced fermentation group, where *Aspergillus* emerged as the dominant fungal species. In contrast, the abundance of Mucoromycota decreased compared to the ordinary fermentation group, which may be attributed to the competitive advantage of *Aspergillus niger* in resource acquisition, highlighting its profound impact on the environment.

We further investigated the interactions among microorganisms during the fermentation process through *Aspergillus niger-*enhanced fermentation, revealing a significant increase in the abundance of *Bacillales* and *Pseudomonas*. These bacteria influenced the flavor compounds in the Pu-erh tea through their metabolic activities, with the interactions between *Bacillales* and *Pseudomonas* potentially serving as a key driving factor behind the changes in flavor compounds during *Aspergillus niger*-enhanced fermentation. *Bacillales* are a group of Gram-positive bacteria that produce resistant spores, which are widely found in soil, water, and air [23]. *Bacillales* can promote the rapid decomposition of starch in the fermentation system and produce organic acids and aroma substances such as diacetyl, as well as improve the antioxidant capacity of the finished fermentation product [24,25]. Moreover, *Bacillus* is also an important biopreventive bacterium, which produces a variety of biopreventive metabolites during the growth process [26,27]. After the enhanced fermentation of *Aspergillus niger*, the relative abundance of *Bacillus* increased significantly, and combined with the metabolomic analysis of the flavor substances, the content of amino acid metabolites was significantly increased in the PEPT. The content of amino acid metabolites was significantly higher, and amino acid-related metabolic pathways were significantly up-regulated. *Pseudomonas* is a nucleated Gram-negative *bacillus* with low nutritional requirements, which exists in large quantities in the inter-root zone of tea trees [28]. *Pseudomonas* has the function of decomposing cellulose and starch to produce glucose and breaking down proteins to produce amino acids [20,29]. This finding aligns with previous studies, suggesting that the increase in tea polysaccharide content during post-fermentation may be attributed to the heightened abundance of *Pseudomonas*. *Pseudomonas* not only degrades complex organic compounds but also produces a variety of volatile compounds, and its increased abundance may directly influence the aroma components of the tea. The results showed that the relative abundance of *Pseudomonas* increased, and the content of amino acid metabolites was elevated after enhanced fermentation by *Aspergillus niger*, with a consistent trend, which provided a certain basis for the change in flavor substances in the PEPT.

### 3.2. Enhanced Fermentation by Aspergillus Niger Significantly Alters Composition of Amino Acid Metabolites in Tea Samples

Metabolites in the post-fermentation process of Pu-erh tea are mainly produced by microorganisms in the fermentation process [20]. During the fermentation process, many microorganisms produce specific enzymes that generate distinct flavor compounds. It has been found that *Aspergillus niger* has a promoting effect on the production of sugars and phenolics during the post-fermentation of Pu-erh tea [30], but its effect on amino acids is not clear.

*Aspergillus niger* is a highly enzymatic fungus known for its significant secretion of enzymes, particularly during fermentation, where its secreted proteases exhibit remarkable activity. For instance, *Aspergillus niger* is capable of secreting a substantial amount of acidic proteases in the fermentation environment, which efficiently hydrolyze proteins into peptides and amino acids under low pH conditions. Other hydrolytic enzymes secreted by *Aspergillus niger*, such as glutaminase and aspartase, can further degrade proteins into free amino acids [31]. *Aspergillus niger* not only releases amino acids through protein degradation but may also generate additional amino acids via its secondary metabolites and fermentation metabolic pathways [32]. This phenomenon is akin to the commonly observed mechanisms of amino acid accumulation in fermented foods [33] and has significant implications for the flavor and quality of tea.

In this study, L-alanine, L-lysine, acetylglutamine, L-thyronine, and glycerol-L-proline were found to be the major differential metabolites in the enhanced fermentation of Pu-erh tea by *Aspergillus niger*, and the accumulation of amino acids by *Aspergillus niger* may play a dominant role throughout the post-fermentation process. *Aspergillus niger* has been reported to secrete enzymes that can degrade plant polysaccharides during Pu-erh tea fermentation, and the resulting monosaccharides enter glycolysis to supply energy for amino acid metabolism [22]. Ref. [34] found that protein biosynthesis and degradation may be the main reason for the decrease and increase in amino acid content during tea fermentation under the action of *Aspergillus niger*. The metabolic pathways of L-lysine biosynthesis and degradation in Pu-erh tea fermented by *Aspergillus niger* were significantly up-regulated. L-lysine has the effect of promoting enzyme activity to accelerate fermentation during the fermentation process of tea, and the production of tea polyphenols in tea has a close relationship with L-lysine; the differences in the metabolic pathways of L-lysine in the fermentation samples may be due to the differences in the microbial abundance of L-lysine in the fermentation sample. This experiment indicates that the addition of *Aspergillus niger* during enhanced fermentation altered the abundance of *Bacillales* and *Pseudomonas* in the tea samples thereby influencing the regulation of amino acid metabolic pathways and ultimately affecting the quality of Pu-erh tea.

To further elucidate the relationship between metabolic products and flavor, as well as the underlying formation mechanisms, we conducted a KEGG analysis. This analysis revealed that the primary metabolic pathways in *Aspergillus niger*-enhanced fermented Pu-erh tea are lysine biosynthesis (ath00300) and degradation (ath00310). During the fermentation process, the balance between lysine synthesis and degradation may be disrupted, leading to increased lysine production, which aligns with the previous observation that L-lysine is a major differential metabolite in *Aspergillus niger*-enhanced fermented Pu-erh tea. Lysine is an umami amino acid, and an increase in its concentration may enhance the mouthfeel of Pu-erh tea. Additionally, some lysine is degraded to produce new metabolic products, including metabolic intermediates such as acetyl-CoA [35], which may subsequently enter the tricarboxylic acid (TCA) cycle or participate in fatty acid synthesis [36] thereby partially explaining the variations in fatty acid compounds during fermentation. The biosynthesis pathway of valine, leucine, and isoleucine (ath00290) indicates that the metabolic intermediates produced from these branched-chain amino acids (BCAAs) during *Aspergillus niger* fermentation, particularly α-keto acids, may be converted into alcohols, ketones, and other volatile flavor compounds [37], directly influencing the aroma characteristics of fermented tea; the metabolic product of isoleucine, isovalric acid, imparts fruity or floral notes to the tea [38]. The fermentation process involving *Aspergillus niger* enhances the biosynthesis of these branched-chain amino acids further impacting the flavor and quality of the tea. The phenylpropanoid biosynthesis pathway (ath00940) involves the conversion of phenylalanine into secondary metabolites such as coumarin, lignin, and anthocyanins via the action of phenylalanine ammonia-lyase (PAL). The fermentation process involving *Aspergillus niger* may enhance the accumulation of phenylalanine thereby promoting the synthesis of phenylpropanoid compounds, which augment the antioxidant capacity of tea and improve the color and aroma of fermented Pu-erh tea [39]. Phenylalanine serves as a precursor in the Maillard reaction, contributing to aroma development [40]; thus, the enrichment of the phenylpropanoid pathway elucidates the increase in aromatic and flavor compounds in fermented Pu-erh tea. The aminoacyl-tRNA biosynthesis pathway (Aminoacyl-tRNA biosynthesis—ath00970) involves the conjugation of amino acids with their corresponding tRNA, representing a crucial step in protein synthesis mediated by aminoacyl-tRNA synthetase [41]. This enrichment indicates enhanced protein synthesis activity during the fermentation process. Concurrently, *Aspergillus niger* fermentation promotes the degradation of proteins to release amino acids. The dynamic balance between protein synthesis and degradation leads to the generation and accumulation of free amino acids. Additionally, the aminoacyl-tRNA synthesized during this process can reutilize these amino acids for the biosynthesis of secondary metabolites, consequently influencing the flavor-related secondary metabolites in tea.

## 4. Materials and Methods

### 4.1. Experimental Materials

Yunnan Big-leaf Sun-dried Green Tea: produced in Wuliang Mountain, Jingdong Yi Autonomous County, Pu’er City, Yunnan Province; *Aspergillus niger* (MT584291): isolated, purified, identified, preserved, screened, and expanded by Pu’er Tea Research Institute.

### 4.2. Instruments and Equipment

Ultra-high performance liquid chromatograph (1290 UPLC): Agilent Technologies, Santa Clara, CA, USA; high-resolution mass spectrometer (Triple TOF 6600): Shanghai Aibao Caixiang Analytical Instrument Trading Co. JXFSTPRP24, Shanghai, China): Shanghai Jingxin Technology Co., Ltd. (Shanghai, China); ultrasonic instrument (PS-60AL): Shenzhen LeiDeBang Electronics Co. (Shenzhen, China).

### 4.3. Isolation and Identification of Aspergillus Niger

A 10 g sample of Pu-erh tea was added to 90 mL of sterile water and shaken for 20 min to evenly distribute microorganisms in the suspension, resulting in a 10^−1^ dilution. A 10^−5^ dilution was then prepared, and 100 μL of the diluted sample was spread evenly onto Potato Dextrose Agar (PDA) plates. The plates were incubated in an inverted position at 28 °C for 72 h. Colonies exhibiting the typical morphological characteristics of *Aspergillus niger* (e.g., black spore heads, hyaline hyphae) were selected and transferred to fresh PDA plates for purification. The conidiophores and spores were confirmed using microscopy. The purified microorganisms were then used to prepare a spore suspension for PCR amplification. The successfully amplified PCR products were sent to Sangon Biotech (Shanghai, China) Co., Ltd. for sequencing analysis under icebox conditions. The returned sequence results were uploaded to the BLAST tool on the NCBI website (www.ncbi.nlm.Nih.gov) (accessed on 5 July 2019) where strains with 100% sequence similarity to the target sequence in the database were identified as the desired strain.

### 4.4. Tea Fermentation

Using a small sprayer, *Aspergillus niger* was evenly sprinkled on the sun-dried green tea in the ratio of 1:10 for the solid state fermentation of Pu-erh tea, repeated 3 times, controlling the temperature of the fermentation pile at 40–60 °C, humidity at 35%, fermentation environment temperature at 25 °C, environmental humidity at 80%, and turning the heap 6 times, with a total of 55 days of fermentation.

### 4.5. Determination of Metabolic Components

Weigh 20 mg of sample, add 1000 μL of extraction solution (methanol: acetonitrile: water = 2:2:1 (*v*/*v*), containing internal standard 2 μg/mL); vortex mixing for 30 s, add the steel beads, grind at 35 Hz for 4 min, ultrasound for 5 min in an ice-water bath; repeat the grinding and ultrasound treatment 2 times; minus 40 °C static for 1 h; sample centrifuged at 4 °C at 10,000 rpm for 15 min. Then, 500 μL of the supernatant was taken into an EP tube, dried under vacuum, and re-solubilized by adding 400 μL of 50% acetonitrile, vortexed for 30 s, and sonicated for 10 min on an ice-water bath; the sample was centrifuged at 4 °C for 15 min at 13,000 rpm, and the sample was detected by the machine by taking 75 μL of the supernatant into the injection vial. The liquid chromatographic conditions were as follows: column: Waters ACQUITY UPLC BEH Amide (2.1 × 100 mm, 1.7 μm); mobile phase: phase A was an aqueous phase containing 25 mmol/L ammonium acetate and 25 mmol/L ammonia, and phase B was acetonitrile; 0~0.5 min, 95% B; 0.5~7 min, 95~65% B; 7~8 min, 65~40% B; 8~9 min, 40% B; 9~9.1 min, 40~95% B; and 9.1~12 min, 95% B. The flow rate of the mobile phase was 0.5 mL/min, the column temperature was 25 °C, the temperature of the sample plate was 4 °C, and the injection volume was 1 μL for positive ions and 1 μL for negative ions. High-resolution mass spectrometry (HRMS) data acquisition was performed in IDA (information-dependent acquisition) mode. In IDA mode, the data acquisition software (Analyst TF 1.7, AB Sciex) automatically selects ions and acquires their secondary mass spectra based on the primary mass spectral data and predefined standards. The 12 strongest ions with intensities greater than 100 were selected for secondary mass spectrometry scanning in each cycle, with a collision-induced dissociation energy of 30 eV and a cycle time of 0.56 s. The ion source parameters were as follows: GS1: 60 psi; GS2: 30 psi; CUR: 35 psi; TEM: 600 °C; DP: 60 V; and ISVF: 5000 V (Pos)/−4000 (Neg).

### 4.6. Determination of Microbial Diversity

Bacterial genomic DNA was extracted from mixed soil samples of the inter-root soils of six tea gardens using the PowerSoil^®^ DNA Isolation Kit (QIAGEN (formerly MO BIO Laboratories), Carlsbad, CA, USA) according to the steps provided by the manufacturer, and the quality of DNA extraction was detected by 1% agarose gel electrophoresis. The total extracted DNA was used as a template to continue the amplification of the target gene fragments [16], and the amplification primers for the bacterial 16S rRNA gene were Bac 27f (5′-AGAGTTTGATCCTGGCTCAG-3′) and Bac 1492r (5′-ACGGCTACCTTGTTACGACTT-3′); the amplification primers for the fungal region of ITS were ITS1F (5′-CTTGGTCATTTAGAGGAAGTAA-3′) and ITS2 (5′-GCTGCGTTCTTCATCGATGC-3′). The PCR reaction was carried out on a PCR automated amplifier, and according to the primers and Taq enzyme used, the annealing temperature was selected to be 55 °C, the extension temperature was selected to be 72 °C, and the number of cycles was selected to be 34. The specific reaction procedures were as follows: (1) 94 °C for 5 min; (2) 94 °C for 30 s, 55 °C for 30 s, 72 °C for 90 s; (3) 72 °C extension for 10 min; and 4 °C unlimited heat preservation. The purified PCR products were subjected to DNA library construction and high-throughput sequencing using the Illumina Miseq platform (HiSeq 2500) of Shanghai BioBioMedical Technology Co. (Shanghai, China).

### 4.7. Non-Target Metabolomics Data Analysis

ProteoWizard was used to process mass spectrometry data for metabolite analysis; multivariate statistical analysis was used to initially screen out the different metabolites among different samples based on the orthogonal projections to the latent structures discriminant analysis (OPLS-DA) results, and then further screened by combining the *p*-value or multiplicative value of the univariate analysis; the differential metabolites were screened by combining the fold change (FC) and the variable important in proiection (VIP) values of the OPLS-DA model variables. The following was the screening criteria: metabolites with FC > 2 (up-regulation) and FC < 0.5 (down-regulation) were selected, and metabolites with a VIP greater than 1 were considered to be significantly different.

### 4.8. Amplicon Sequencing Data Analysis of 16S rDNA 

Sequence splicing was performed with FLASH v1.2.7 software for bipartite sequencing, Trimmomatic v0.33 software for filtering Raw Tags, and UCHIME v4.2 software for removing chimeras. The Tags with similarity at 97% level were clustered, OTUs (operational taxonomic units) were obtained using Usearch software (V9), and the feature sequences were taxonomically annotated by using SILVA as the reference database. The dilution curves were plotted by using the index calculated by QIIME (V2.2023) with the software R (V4.3.2). Alpha Diversity analysis of the samples was carried out by using the Mothur software (V1.40.5), and based on the results of the OTUs, the Shannon index, Chao1 index, etc., the dilution curves of the samples were plotted. The Beta Diversity analysis of the samples was performed by using Qiime (Version 1.7.0) software to explore the differences between the samples.

### 4.9. Association Analysis

Based on the Illumina double-end sequencing platform and the selected mutated regions, we designed universal primers for PCR amplification by using the conserved regions, and sequenced the highly mutated regions for the OTUs (operational taxonomic units) sequence analysis and strain identification. The β-diversity was used for intergroup analysis to identify species with significant changes in abundance between groups at different taxonomic levels. Qualitative and relative quantitative analysis of the small molecule metabolites in the biological samples based on LC-MS chromatography-mass spectrometry. The VIP value of OPLS-DA and the *p*-value of univariate statistical analysis were used to identify the metabolites with significant changes in abundance among subgroups. The correlation coefficients between the significantly different flora and the significantly different metabolites in the samples were analyzed by using spearman statistics and combined the R language and Cytoscape software (V3.x) to explore the interaction relationship between the flora and metabolites from multiple angles.

### 4.10. Statistical Methods

ANOVA one-way significance analysis was performed using SPSS 27.0 statistical software, denoted by SD; graphs were plotted by using Graphpad prism 9.0.2. *p* < 0.05 indicates a statistically significant difference, and *p* < 0.01 indicates a highly significant difference.

## 5. Conclusions

This study explored how *Aspergillus niger* inoculation in sun-dried green tea enhances fermentation and influences the microbial community and metabolite composition of Pu-erh tea. Enhanced fermentation resulted in notable shifts in the metabolite profile, with 95 metabolites significantly increasing and 105 decreasing, with amino acids showing the most substantial changes. High-throughput sequencing revealed no significant alterations in the fungal community but highlighted a marked increase in bacterial populations such as *Bacillales* and *Pseudomonas* in the presence of *Aspergillus niger*. These changes fostered a more balanced and interactive microbial ecosystem, driving the production of amino acids and related metabolites, which ultimately enhanced the flavor and overall quality of the Pu-erh tea.

The findings highlight the significant potential of *Aspergillus niger* in optimizing the fermentation process of Pu-erh tea, offering valuable insights into the functional roles of microorganisms in fermented foods. Future research should aim to uncover the dynamic interactions between *Aspergillus niger* and other microorganisms, refine fermentation parameters, and apply multi-omics approaches to identify key enzymes and metabolic pathways. These efforts could enhance fermentation efficiency and improve product quality. Additionally, broadening the use of *Aspergillus niger* in tea production and exploring its applications in other fermented foods could open new avenues for commercialization, delivering high-value fermentation solutions to the food industry.

## Figures and Tables

**Table 1:** Alpha diversity indices of bacteria and fungi of samples from different fermentation treatments.

Samples	Richness Index				Diversity Index			
	ACE		Chao1		Simpson		Shannon	
	Bacteria	Fungi	Bacteria	Fungi	Bacteria	Fungi	Bacteria	Fungi
CK	430.89 ± 9.75 ^a^	207.27 ± 7.33 ^a^	440.44 ± 6.56 ^a^	207.00 ± 7.23 ^a^	0.22 ± 0.06 ^a^	0.10 ± 0.00 ^a^	2.96 ± 0.33 ^a^	3.22 ± 0.06 ^a^
NF	383.26 ± 35.58 ^a^	62.03 ± 17.32 ^b^	393.79 ± 35.07 ^a^	35.12 ± 2.77 ^b^	0.05 ± 0.01a ^b^	0.39 ± 0.04 ^a^	3.78 ± 0.14 ^b^	1.01 ± 0.06 ^b^
TF	285.51 ± 25.59 ^b^	130.14 ± 25.82 ^c^	296.14 ± 33.15 ^b^	104.26 ± 39.57 ^b^	0.10 ± 0.01 ^b^	0.45 ± 0.03 ^b^	2.85 ± 0.10 ^b^	0.93 ± 0.05 ^b^

Lower letters ^a,b^, and ^c^ represents a significant difference.

## Data Availability

Data are contained within the article and Supplementary Materials.

## Supplementary Materials

The following supporting information can be downloaded at: https://www.mdpi.com/article/10.3390/molecules29235647/s1, Table S1: CK, NF, and TF groups’ metabolites. Table S2: Differential metabolites between the NF and TF groups.
